# Degradable silk fibroin based piezoresistive sensor for wearable biomonitoring

**DOI:** 10.1186/s11671-024-04001-z

**Published:** 2024-03-25

**Authors:** Chunlin Pang, Fei Li, Xiaorao Hu, Keyu Meng, Hong Pan, Yong Xiang

**Affiliations:** 1 School of Materials and Energy, University of Electronic Science and Technology of China, Chengdu, 611731 China; 2 School of Electronic and Information Engineering, Changchun University, Changchun, 130022 China; 3 Advanced Energy Institute, University of Electronic Science and Technology of China, Chengdu, 611731 China; 4 Sichuan Flexible Display Material Genome Engineering Center, Chengdu, China; 5 Tianfu Jiangxi Laboratory, Chengdu, 610041 China

**Keywords:** Degradable, Silk fibroin, Hollow carbon spheres, Wearable, Biomonitoring

## Abstract

Degradable wearable electronics are attracting increasing attention to weaken or eliminate the negative effect of waste e-wastes and promote the development of medical implants without secondary post-treatment. Although various degradable materials have been explored for wearable electronics, the development of degradable wearable electronics with integrated characteristics of highly sensing performances and low-cost manufacture remains challenging. Herein, we developed a facile, low-cost, and environmentally friendly approach to fabricate a biocompatible and degradable silk fibroin based wearable electronics (SFWE) for on-body monitoring. A combination of rose petal templating and hollow carbon nanospheres endows as-fabricated SFWE with good sensitivity (5.63 kPa^−1^), a fast response time (147 ms), and stable durability (15,000 cycles). The degradable phenomenon has been observed in the solution of 1 M NaOH, confirming that silk fibroin based wearable electronics possess degradable property. Furthermore, the as-fabricated SFWE have been demonstrated that have abilities to monitor knuckle bending, muscle movement, and facial expression. This work offers an ecologically-benign and cost-effective approach to fabricate high-performance wearable electronics.

## Introduction

The booming advancement of the Internet of things (IoT) and 5G wireless technology promotes widespread application of wearable electronics in various scenarios, such as biomonitoring [1–4], soft-robotics [5, 6], human–machine interfacing [7, 8], and artificial intelligence [9–11]. The ever-growing demand of electronic devices render the massive waste electronic wastes (e-wastes), leading to severely damaging the natural environment and even breaking up the continuous carbon cycle in the whole ecological system [12, 13]. Most seriously, numerous e-wastes contain toxic elements, such as mercury, arsenic, chromium, and lead, inducing serious health problems when e-wastes are casually discarded in the surroundings [14, 15]. According to the World Health Organization predictions, e-wastes are expected to grow further to 747 thousand tons by 2030 following a yearly growth rate of nearly 2 million tons [16]. It is urgent to develop biosustainable and degradable electronics to weaken or eliminate the negative effect of waste e-wastes and promote the development of medical implants without secondary post-treatment. In this regard, sensing materials possess nonpoisonous and degradable nature that can be decomposed into absorbable nontoxic low-molecular compounds, providing an innovative perspective for building environment-friendly and biocompatible electronics.

Natural and synthetic degradable materials such as starch, gelatin, silk, cellulose, polylactic acid (PLA), polyurethane (PU), etc. have been widely explored as building blocks for constructing disposable or transient electronics [17–19]. Among them, silk is an ancient protein fiber along with appealing properties including biocompatibility, biodegradability, bioresorbability, and natural abundance, have been served as excellent candidates for the fabrication of bioelectronic [20–22]. However, silk fibroin films usually have low toughness, poor elasticity, and mechanical mismatch with skin, which hinders further integration. In recent, calcium-modified silk fibroin was demonstrated that have been plasticized into soft and stretchable films and capable of conformal contact with skin, which offer an efficient approach to construct wearable and implantable silk fibroin-based electronics [23]. Besides that, both highly sensing performances and low-cost manufacture must be taken into consideration. Most reported silk-based wearable sensors incur poor performance (low sensitivity, slow response time, etc.) or high cost (complex fabrication process, large-scale instruments, and pollution, etc.) [24–27]. Thus, rational architectural design and simplified fabrication process are highly desirable in the construction of silk-based wearable electronics.

Herein, we demonstrated a facile, low-cost, and environmentally friendly approach to fabricate biocompatible and degradable silk fibroin based wearable electronics (SFWE) for on-body monitoring. A combination of rose petal templating and carbon hollow nanospheres endows the as-fabricated SFWE with sensitivity of 5.63 kPa^−1^, fast response time of 147 ms, and good mechanical durability (15,000 cycles). In addition, the as-synthesized SFWE show great capability in monitoring physiological activities of knuckle bending, muscle motion, and facial expression. This work offers a novel paradigm for developing high-performance and cost-effective degradable wearable electronics.

## Experimental section

### Preparation of hollow carbon nanospheres (HCNs)

The silica spheres were purchased from Hangzhou Xinqiao Biotechnology Co. Ltd (China). The aminopropyl triethoxysilane (APTES), ethanol, glucose, HF, CaCl_2_, and formic acid (FA) were purchased from Aladdin (Shanghai, China). All chemicals were used as received without further purification.

**Fig. 1:**
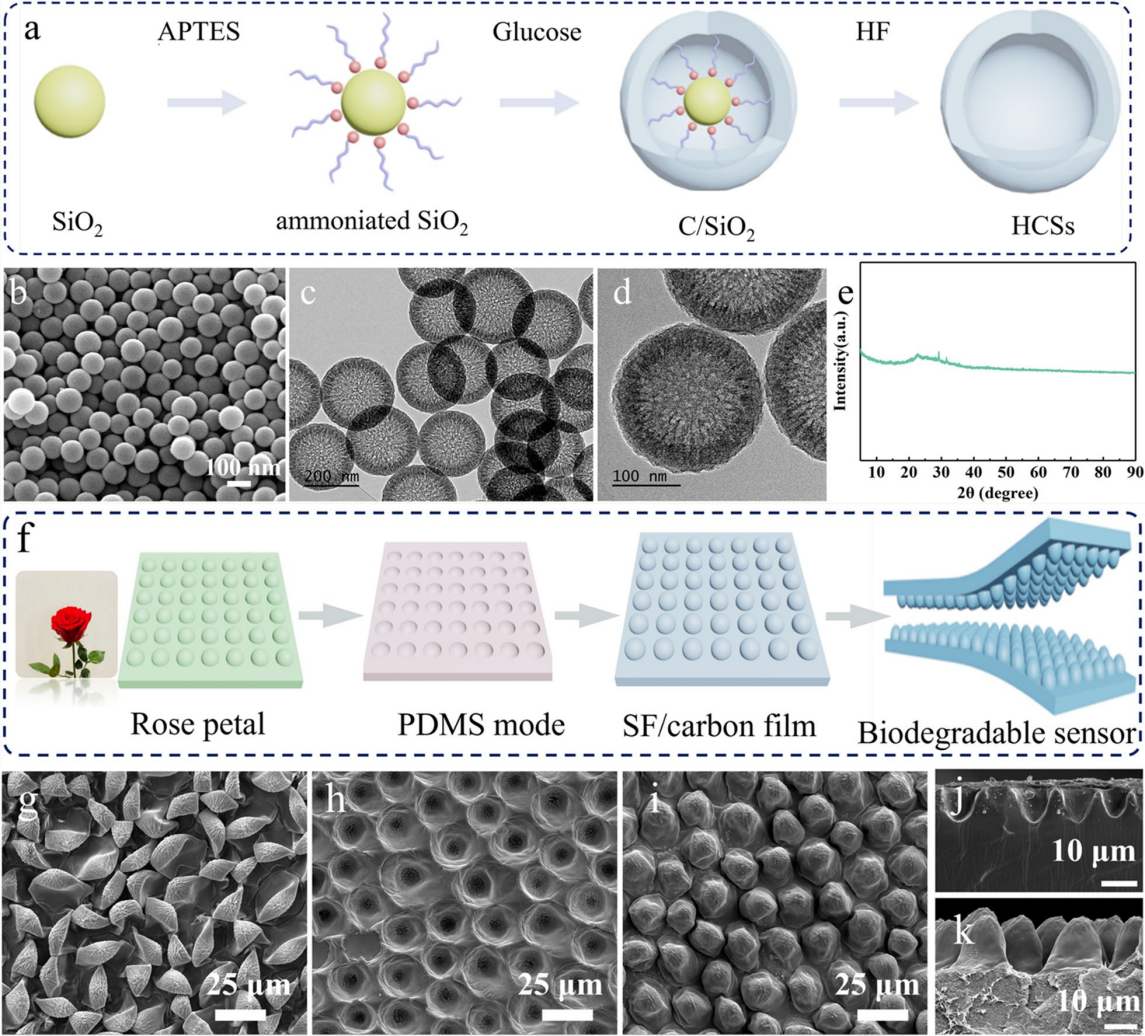
**a** Schemes showing the preparation process of hollow carbon nanospheres (HCNs). **b** The SEM image of hollow carbon nanosphere. **c**–**d** The TEM image of hollow carbon nanosphere. **e** The XRD spectrum of HCNs/silk fibroin. **f** Schemes of the fabrication process for microstructural silk fibroin based piezoresistive sensors. The SEM images of **g** rose petal, **h** microstructural PDMS films, and **i** microstructural HCNs/silk fibroin composite films. The cross-sectional SEM images of **j** microstructural PDMS films and **k** microstructural HCNs/silk fibroin composite films

The hollow carbon spheres were synthesized using ammoniated silica spheres as a core template and glucose as carbon source [28], the detailed fabrication process as shown in schematic illustration (Fig. 1a). Firstly, silica spheres were ammoniated with APTES to strengthen the adhesion between silica spheres and carbon source. Briefly, silica spheres (1.5 g) were added into a solution containing 2.5 mL APTES and 25 mL ethanol, ultrasonic treatment for 1 h followed by stirring 3 h at room temperature to fulfil the ammoniated treatment, and then obtained suspension was washed with deionized water (DI water) and centrifuged several times, the obtained sediment was dried at 45 °C for 24 h in the vacuum oven. Subsequently, the ammoniated silica spheres were obtained.

To fabricate HCNs, glucose (1 g) and as-obtained ammoniated silica spheres (0.12 g) were added into 10 mL DI water followed by ultrasonic treatment for 1 h, the mixture was transferred to a Teflon lined autoclave and treated at 180 °C for 9 h. The obtained suspension was washed with deionized water and ethanol several times and dried at 80 °C for 24 h in the vacuum oven. Followed by heat treatment at 900 °C for 2 h in the nitrogen atmosphere, the obtained carbon wrapped silica composite was immersed into 4 M (hydrogen fluoride) HF solution for 12 h to etch out SiO_2_ nanospheres, and then dried at 45 °C for 24 h. Finally, the hollow carbon nanospheres were successfully synthesized.

### Fabrication of silk fibroin based piezoresistive sensors

The fresh rose petals were washed with DI water for three times and then cut into a rectangle. After dried by air blowing, the rose petal rectangle was fixed on a polyethylene (PE) petri-dish using tapes. The polydimethylsiloxane (PDMS, Sylgard 184, Dow Corning) prepolymer was prepared in a 1: 8 w/w ratio of curing agent to elastomer and placed in a vacuum chamber at 25 °C for 40 min until bubbles disappeared. Subsequently, the PDMS prepolymer was coated on the rectangular rose petal, followed by natural curing at 25 °C for three days. The cured PDMS was peeled off as a second molding template.

CaCl_2_ (0.6 g) was dissolved in 10 mL FA, followed by adding scissored silk fibroin (1.2 g) into above-mentioned solution, stirring for 4 h to form silk fibroin transparent solution. Subsequently, HCNs (0.12 g) were added to silk fibroin solution, followed by stirring for 2 h. The HCNs/silk fibroin films were obtained by coating mixed solution on the microstructural PDMS films and then placed in a fume hood to remove FA from the HCNs/silk fibroin films. The as-fabricated HCNs/silk fibroin films were peeled off and cut to desired shapes. After these two-molding process, a replica of the original surface, namely, the HCNs/silk fibroin films with positive topology were fabricated, serving as sensing layer. The piezoresistive pressure sensor (with the size of 1 cm × 1 cm) was fabricated by face-to-face assembling two layers of microstructural HCNs/silk fibroin films, and silver paste was coated on one edge of each HCNs/silk fibroin film to serve as electrodes. Finally, the device was packaged with medical PU tape.

### Characterizations and measurement

The morphologies of the HCSs/silk fibroin film were characterized by a field-emission scanning electron microscope (FESEM, FEI Inspect F50, USA). The composition was measured by X-ray diffraction (DX-100, Dandong haoyuan instrument Co., Ltd., China). The Fourier transform infrared spectroscopy (FTIR, Nicolet 6700, Thermo Fisher, USA) and an X-ray photoelectron spectrometer (XPS, Thermo Scientific K-Alpha, USA) were applied to analyze the original sample and after degradable sample. An electric vertical test stand (SJS-500, SHANDU) was utilized to load the external pressure on the devices, along with a digital force gauge (SH-50, Wenzhou Sundoo Instruments Co., Ltd., China) was applied to measure the value of the loading pressure. The electromechanical characteristics of piezoresistive sensors was measured by the Keithley 4200 SCS.

## Results and discussion

Figure 1a illustrates the fabrication process of hollow carbon nanospheres using ammoniated silica spheres as a core template and glucose as carbon source. Briefly, silica nanospheres were ammoniated introducing APTES to strengthen the adhesion between the silica and carbon source. Subsequently, utilizing glucose as carbon source and combination of hydrothermal reaction to prepare C/SiO_2_, and using HF etch out SiO_2_ to obtain hollow HCNs; the morphology of HCNs is shown in Fig. 1b–d. Figure 1b shows the SEM image of HCNs, which exhibit a spherical structure with uniform size distribution and an average diameter of approximately 200 nm. The TEM images were measured to further explore inner structure of carbon nanospheres, as shown in Fig. 1c, d, it’s clear that the carbon nanosphere is composed of a hollow white part and a dark spherical ring, indicating that SiO_2_ has been completely removed and hollow carbon nanosphere has been successfully fabricated. From the enlarged TEM image of HCNs, the thickness of the carbon spheres is about 20 nm. The XRD pattern was measured to investigate the influence of HCNs on the structure of silk fibroin. As shown in Fig. 1e, the spectrum of composite film has only one diffraction peak at 2θ = 20.4°, which ascribed to the *β*-sheet structure of silk fibroin, indicating that the addition of HCSs has no effect on the structure of silk fibroin [29, 30]. Figure 1f illustrates the fabrication process of silk fibroin based piezoresistive sensors. Briefly, two layers of microstructural HCNs/silk fibroin films were placed face-to-face to construct piezoresistive sensors. The microstructural HCNs/silk fibroin film with positive topology was fabricated through two-molding process from a piece of fresh rose petal. Figure 1g exhibits continuous microdome structures on the surface of the fresh rose petal. Utilizing fresh rose petals as the mold, large area PDMS films with uniform hierarchical microstructures could be obtained, as shown in Fig. 1h. Followed by using as-obtained microstructural PDMS films as the mold to construct HCNs/silk fibroin film with positive topology of the fresh rose petal, the corresponding morphology is shown in Fig. 1i. The cross-sectional SEM images of microstructural PDMS films and microstructural HCNs/silk fibroin composite films were further confirm that HCNs/silk fibroin films with positive topology of the fresh rose petal have been successfully prepared, as shown in Fig. 1j, k, and the convex height of the microdome is about 120 µm.

**Fig. 2:**
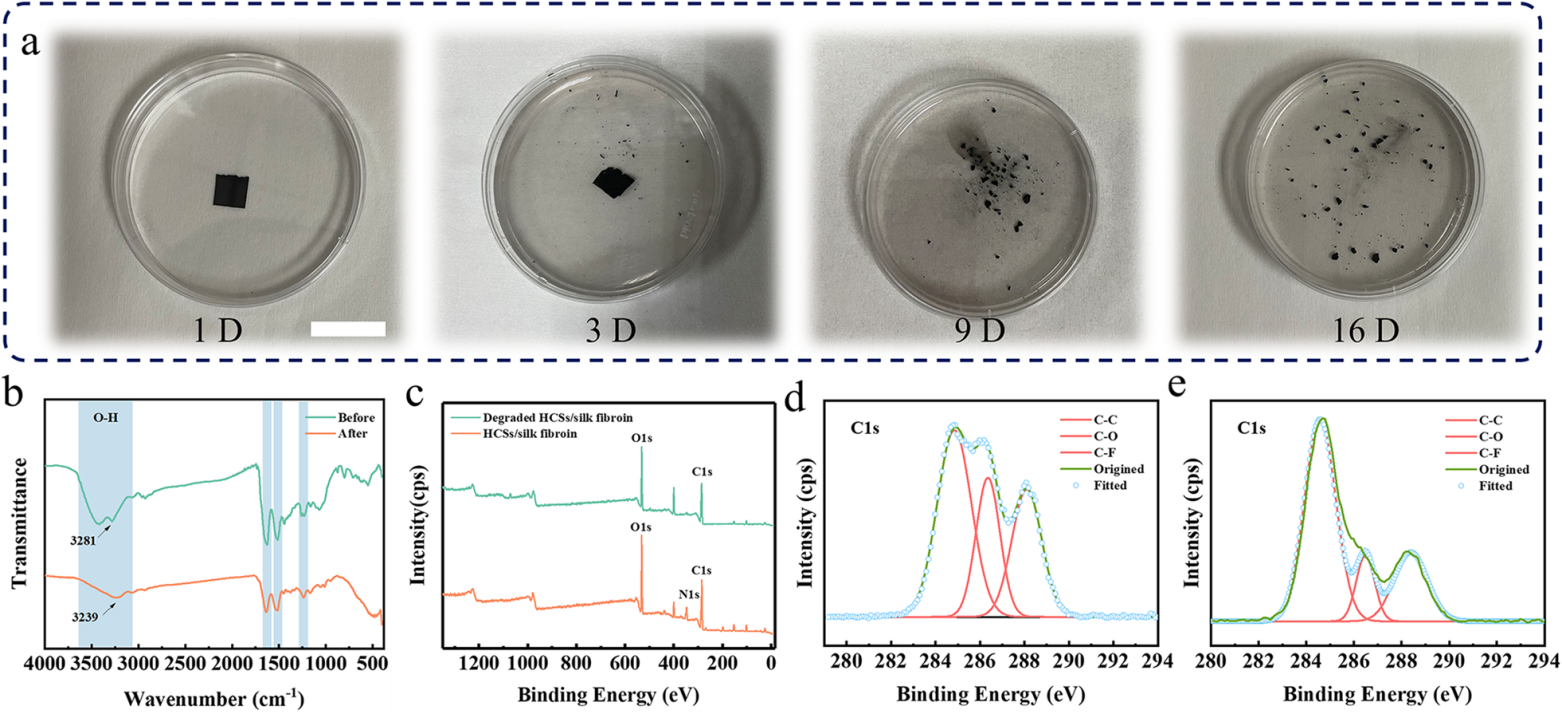
**a** Photographs of HCNs/silk fibroin composite films placed in a 1 M NaOH solution for different times, scale bar: 3 cm. **b** FTIR patterns of before and after degradable HCNs/silk fibroin composite films. **c** XPS survey spectra and C 1s spectra of the **d** HCNs/silk fibroin composite films and **e** degraded HCNs/silk fibroin composite films

Silk fibroin based piezoresistive sensors (SFPS) were composed of interlocking two HCNs/silk fibroin films, thus exploring the degradable characteristics of SFPS is equivalent to evaluating the degradable performance of HCNs/silk fibroin films. As shown in Fig. 2a, the composite film was placed into 1 M NaOH solution at 25 °C. After 16 days of preservation, the film slowly changes into fragments and the size becomes shrinks, indicating that as-prepared HCNs/silk fibroin film was degraded. To further analyze the degradable characteristics of HCNs/silk fibroin film, the FT-IR spectra of HCNs/silk fibroin film before and after degradation (Fig. 2b). The peaks at 1627–1636 cm^−1^, 1513–1515 cm^−1^, and 1232–1235 cm^−1^ respectively correlate to amide I, II, and III of the secondary structure of silk fibroin (silk-II), where amide I and II are assigned to *β*-sheet conformation, and amide III is assigned to random coil or helical conformation. The peak at 3235–3281 cm^−1^ is assigned to the stretching vibration of –OH group of silk fibroin [31, 32]. In comparison to HCNs/silk fibroin film before degradation, the apparent decrease in intensity of the *β*-sheet conformation in secondary structures (peaks of amide I, and II) of HCNs/silk fibroin film after degradation in the pattern of hybrid fibers [33]. The results indicated that the decreased in content of β-fold structure after degradation, indicating that HCNs/silk fibroin composite film was degraded [34, 35].

Furthermore, the XPS spectrums of original and degraded HCNs/silk fibroin were measured to further verify the degradability feature of silk fibroin based piezoresistive sensors (SFPS). In the XPS survey spectrum (Fig. 2c), the original and degraded HCNs/silk fibroin demonstrated the characteristic peaks at 284.6 eV (C 1s), 399.9 eV (N 1s), 531.8 eV (O 1s) [36]. The O/C ratio of degraded HCNs/silk fibroin is 43.2% that lower than the O/C ratio (48.9%) of original HCNs/silk fibroin, indicating the decrease in the oxygen functionalities, which verify the HCNs/silk fibroin film possess the degradability. The C 1s high-resolution peaks fits of the original and degraded HCNs/silk fibroin were utilized to investigate the relationship between carbon and oxygen, hence distinguishing the difference in their functional groups, as plotted in Fig. 2d–e. The C 1s level spectra demonstrated three characteristic peaks, including peak 1 is assigned to the C–H and C–C bonds at 284.6 eV, peak 2 (286.2 eV) corresponding to C–O–C and C–OH bonds, and peak 3 represents –C=O bonds at 288.7 eV [37, 38]. In comparison to original HCNs/silk fibroin film, the peak 2 (C–O–C) and peak 3 (–C=O) in XPS spectrum of degraded HCNs/silk fibroin was distinctly weakened, indicating that the –N–C=O bond was broken, which further confirm that the degradability of HCNs/silk fibroin composite films.

**Fig. 3:**
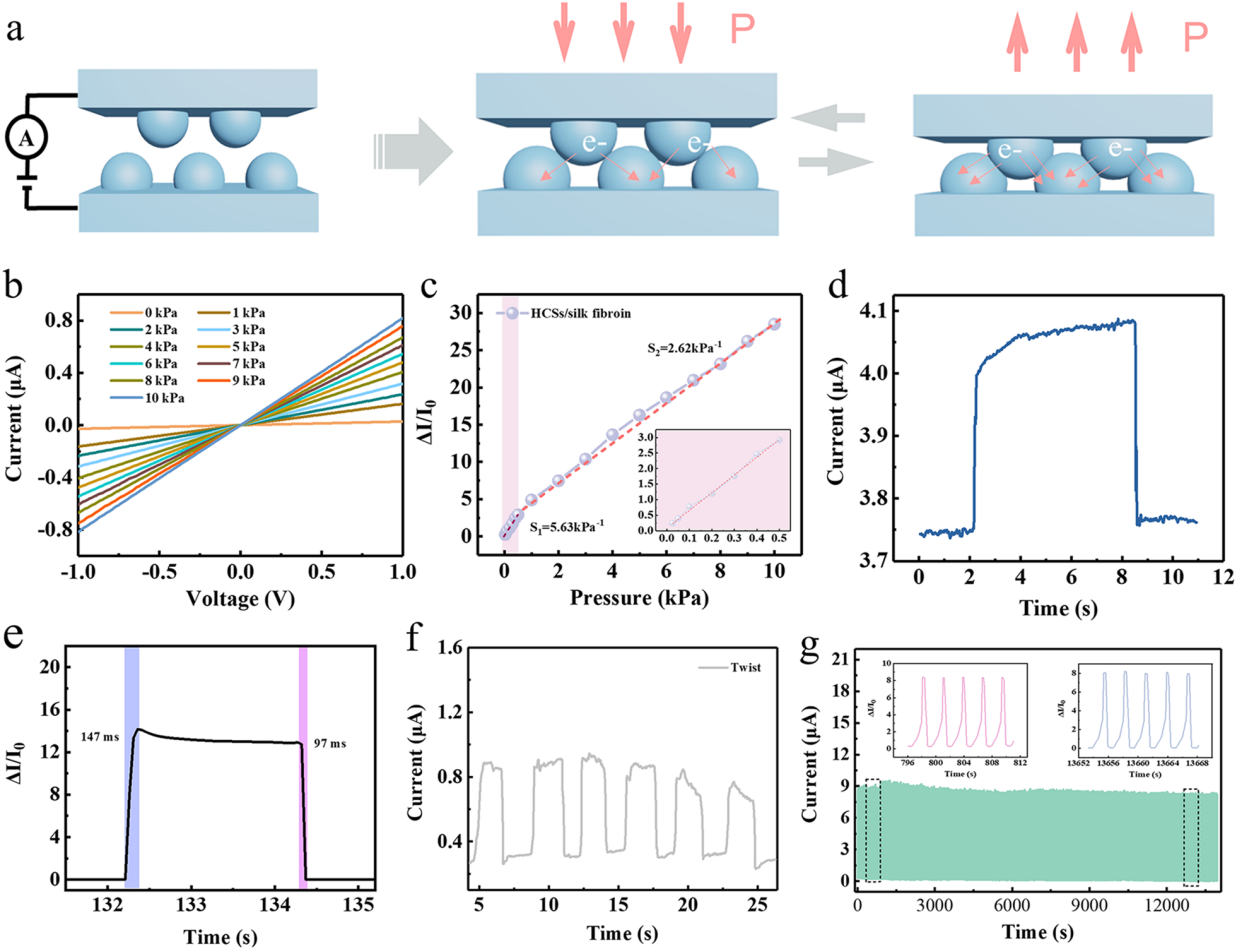
**a** Schematic illustration of the sensing mechanism of degradable HCNs/silk fibroin piezoresistive sensors. **b** The I-V curves of the degradable HCNs/silk fibroin piezoresistive sensors with various pressures from 0 to 10 kPa. **c** The Relative current change versus applied pressure curve for HCNs/silk fibroin piezoresistive sensors. **d** The transient response of pressure sensor to loading and removal of metal sheet (about 1 g), corresponding to a pressure of 0.1 kPa. **e** The response time and recovery time of the sensor. **f** The real-time response of pressure sensors while reversibly twisting the device. **g** Durability test with 15,000 loading/unloading cycles under a constant pressure

A schematic illustration of silk fibroin based piezoresistive sensors (SFPS) is drawn to attain a vivid understanding of the sensing mechanism, as shown in Fig. 3a. The pressure-sensing mechanism of silk fibroin based piezoresistive sensors mostly relies on the geometric effect, where contact area between two microdomes has an impact on the resistance of SFPS [2, 39]. In the initial state, the top and the bottom HCNs/silk fibroin films contact with each other, forming conductive paths. With a pressure applied, the increase in the contact area between two microdomes determine, leading to the increase in conductive paths and current of SFPS. while the applied pressure further increases, the hollow carbon nanospheres are squeezed, resulting in the increase in current of sensors. When the applied pressure is removed, the current will fall to the original state of sensors, which ascribe to excellent flexibility and recoverable deformation of the HCNs/silk fibroin film. As plotted in Fig. 3b, the I–V curves of SFPS were measured with different pressures (0–10 kPa) at a voltage range from − 1 to 1 V, where an excellent linear relationship demonstrates that the HCNs/silk fibroin film and electrodes is the ohmic contact under diverse external pressures, and the slope of I–V curve increases as applying larger pressure is due to the decrease in contact resistance between two HCNs/silk fibroin films. As displayed in Fig. 3c, the curve of relative current change of SFPS versus applied pressure is composed of two varying ranges demonstrating an abrupt increasing trend and slow increasing tendency under relatively high pressures. The abrupt increasing trend can be observed under relatively low-pressure results from the rapid increase in contact area among microdomes. As the pressure increases, the deformation of microdomes tends to be saturated, the comparatively slow change in relative current of these sensors stemmed from the deformation of hollow carbon nanospheres. The SFPS exhibits the linear sensitivity of 5.63 kPa^−1^ in wide detection range from 0.05 to 0.5 kPa and relatively low sensitivity of 2.62 kPa^−1^ in the pressure range from 0.5 to 10 kPa.

To analyze the ability of SFPS to monitor subtle pressure, a metal sheet with a weight of 1 g (corresponding to a pressure of 0.1 kPa) was loaded and unloaded on the SFPS, and then recorded change in current. As displayed in Fig. 3d, a steady and clear output current vibration gives rise to dynamic detection limit as low as 0.1 kPa. Figure 3e presents that the response/recovery time of SFPS is about 147 ms/87 ms. To evaluate the stability of the sensor, as displayed in Fig. 3f, the reversible twisting was applied on the surface of devices, the output current increase and then fall to the initial value, implying good stability and robustness of SFPS. For wearable electronics, good mechanical robustness is highly desired for long-term application. As plotted in Fig. 3g, the output signal did not conspicuously appear to decline by more than 5% during the whole 15,000 cycles under a periodicity pressure of 2 kPa, indicating the SFPS has durable repeatability and stability.

**Fig. 4:**
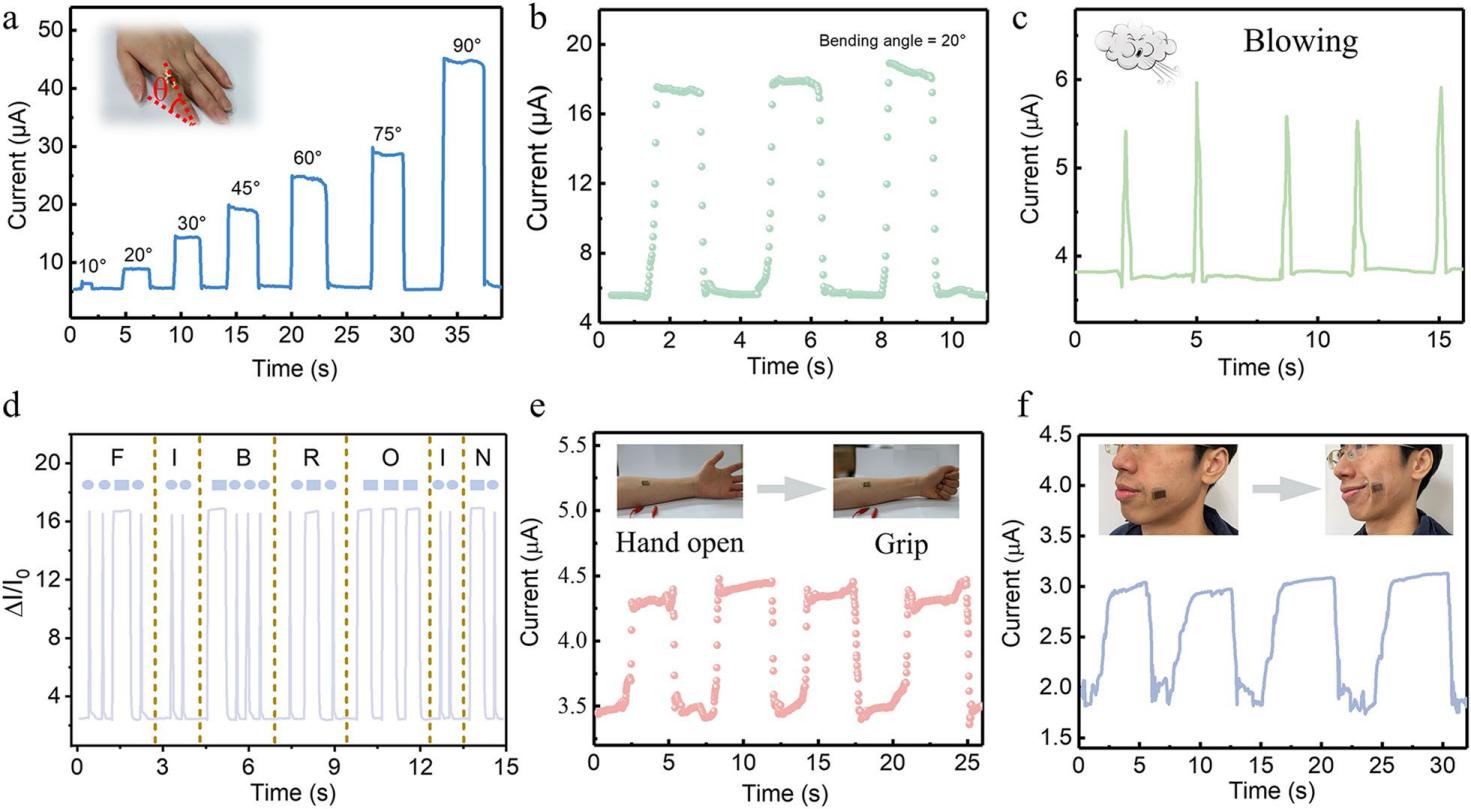
**a** Real-time current change of pressure sensor in response to finger bending with various bending angles from 10°to 90°. Inset: The photograph of finger motions. **b** The real-time current profiles of the device in response to finger bending with bending angle of 20°. **c** Corresponding signals for the process of expiration. **d** The relative current change of Morse code for “fibroin”. **e** Sensing performance of the device attached to the arm muscle for real-time detection of radial muscle contraction. Inset: Photograph of the device attached to the arm muscle in the process of hand open and grip. **f** Real-time current change of pressure sensors in response to the process of grinning

To demonstrate the capability of the SFPS for biomonitoring, the as-fabricated SFPS is attached on the diverse sites of human body to detect physiological activities. The dynamical monitoring finger bending motion were obtained by attaching SFPS to the volunteer’s finger, and then recorded the distinct and steady fluctuation curve of current, as shown in Fig. 4a, the output current increases with increasing bending angle from 10° to 90°. Figure 4b presents three sets of the transient response to finger bending at bending angle at 20°, indicating that the excellent mechanical stretchability and stability of the device. To detect the process of expiration, the SFPS was fixed on the mask and repeatable and steady current signal can be precisely recorded during periodic expiration, as plotted in Fig. 4c. By attaching to the volunteer’s finger-pulp, static and transient pressures were applied on the surface of device to record the Morse code, as displayed in Fig. 4d. Seven types of the vibration curve of current, including flat and/or sharp peak were attained corresponding to seven characters such as “F”, “I”, “B”, “R”, “I”, “O”, and “N”, respectively. The result implies SFPS can be served as a messenger to secretly transfer encrypted information. The SFPS was worn on the arm to monitor the muscle contraction by repeatedly making a fist. As shown in Fig. 4e, the current signal increases while making a fist and then falls to the original state once opening palm, demonstrating the complicated muscle movements can be captured by the SFPS. As shown in Fig. 4f, the SFPS was attached on volunteer’s cheek to record the process of grinning, the distinct and stable vibration curve of current indicates the monitoring capability of SFPS in muscle contraction. These results verify that SFPS has great potential application in physiological status monitoring.

## Conclusions

In summary, we developed biocompatible, biodegradable, and ecologically benign SFPS for wearable biomonitoring via the combination of rose petal templating and carbon hollow spheres. An efficient degradation of SFWE were observed in the solution of 1 M NaOH. FTIR and XPS analysis results further confirm the degradable nature of SFPS. The as-prepared SFPS demonstrates a good sensitivity of 5.73 kPa^−1^, a fast response time, and cyclic stability (15,000 cycles), along with excellent sensing capability to monitor diverse physiological, including finger motion, muscle contraction, and facial expressions, revealing great feasibility in wearable biomonitoring.

## Data Availability

All data generated or analyzed during this study are included in this published article.
